# Risk Factors Associated with Peritoneal-Dialysis-Related Peritonitis

**DOI:** 10.1155/2012/483250

**Published:** 2012-12-20

**Authors:** Julia Kerschbaum, Paul König, Michael Rudnicki

**Affiliations:** Department of Internal Medicine IV (Nephrology and Hypertension), Medical University Innsbruck, Anichstraße 35, 6020 Innsbruck, Austria

## Abstract

*Background*. Peritonitis represents a major complication of peritoneal dialysis (PD). The aim of this paper was to systematically collect data on patient-related risk factors for PD-associated peritonitis, to analyze the methodological quality of these studies, and to summarize published evidence on the particular risk factors. *Methods*. Studies were identified by searches of Pubmed (1990–2012) and assessed for methodological quality by using a modified form of the STROBE criteria. *Results*. Thirty-five methodologically acceptable studies were identified. The following nonmodifiable risk factors were considered valid and were associated with an increased risk of peritonitis: ethnicity, female gender, chronic lung disease, coronary artery disease, congestive heart failure, cardiovascular disease, hypertension, antihepatitis C virus antibody positivity, diabetes mellitus, lupus nephritis or glomerulonephritis as underlying renal disease, and no residual renal function. We also identified the following modifiable, valid risk factors for peritonitis: malnutrition, overweight, smoking, immunosuppression, no use of oral active vitamin D, psychosocial factors, low socioeconomic status, PD against patient's choice, and haemodialysis as former modality. *Discussion*. Modifiable and nonmodifiable risk factors analyzed in this paper might serve as a basis to improve patient care in peritoneal dialysis.

## 1. Introduction

Peritonitis still represents the main acute complication of peritoneal dialysis (PD) and is a leading cause of hospitalization [1], catheter loss, and technique failure [2]. It is also a common cause of death in PD patients [3] and has been described as one of the leading causes of transfer to hemodialysis (HD). The decline of peritonitis rates during the last decades has mostly been achieved by improvements in factors relating PD technique such as the change to plastic bags, the introduction of the Y-set-twin-bag connection system [4]. Despite the significant drop in the peritonitis rates since the 1980 from approximately 6 episodes/patient year [5], the peritonitis rate published in the literature remains constant at approximately 0.35 episodes/patient year. 

To further reduce the risk of morbidity, mortality, and technique failure patient-specific risk factors, which one can divide into modifiable and nonmodifiable, gain more attention in PD patient care. The aim of this paper was to perform a comprehensive collection of published studies on modifiable and nonmodifiable risk factors for PD-associated peritonitis between 1990 and 2012, to assess the methodological quality of the identified studies and to offer an overview of evidence-based patient factors which are associated with an increased risk for peritonitis in PD patients. 

## 2. Materials and Methods

Relevant studies were identified by searches of Pubmed in April 2012, with key words that included “peritonitis,” “peritoneal dialysis,” and “risk factor”. The search was limited to studies with at least 40 patients in human adults in English language, published between 1990 and 2012. In order to provide an unbiased comparison, only studies reporting on peritonitis of any cause, that is, studies which reported data on all peritonitis episodes regardless of underlying germ were included. Hence, studies only reporting on risk factors for fungal or enteric peritonitis were excluded. The term “patient factor” was defined as a modifiable or nonmodifiable factor which is related to the individual. Data extraction was carried out by J. Kerschbaum and reviewed by M. Rudnicki. Studies were assessed for methodological quality using a modified checklist of the STROBE statement [6] (Table 1). For the purpose of this paper, we limited quality assessment to 15 relevant items. Study quality was considered as “acceptable” if the score was ≥10.

## 3. Results

**Figure 1:**
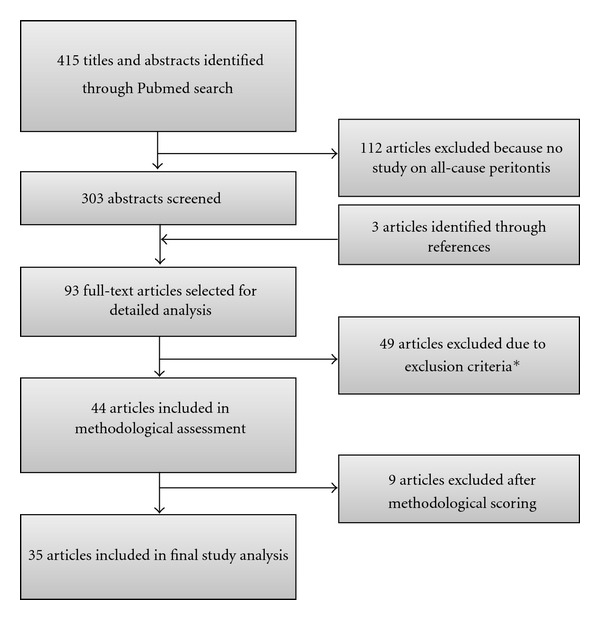
Process of identification of eligible studies. *16 studies: not on all-cause peritonitis; 21 studies: no patient risk factors, 6 studies: cohorts including children; 5 studies: cohorts < 40 patients; 1 study: single event report.

**Figure 2:**
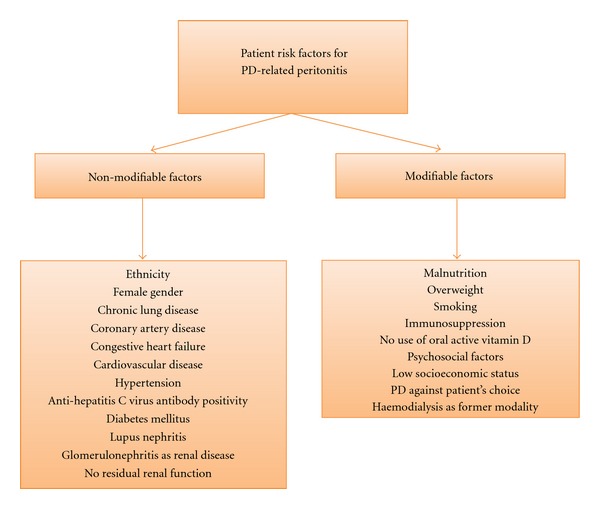
Identified patient risk factors. Factors are divided by nonmodifiable and modifiable risk factors.

The search identified 415 potentially relevant studies. First, 112 articles had to be excluded because they were no study on peritonitis of any cause. Then, 303 abstracts were screened and 3 articles were additionally identified through the references of the former identified articles. In a next step, 93 full-text articles were selected for detailed analysis, 49 articles had to be excluded due to the predefined exclusion criteria. Finally, 44 studies were assessed for methodological quality. Nine articles were excluded because of having low methodological scoring and finally, thirty-five studies were scored as having “acceptable” methodological quality. Selection process is depicted in Figure 1, characteristics of studies are shown in Table 2. Risk factors were divided into nonmodifiable and modifiable factors, a brief overview is shown in Figure 2.

### 3.1. Nonmodifiable Risk Factors (Table 3)

#### 3.1.1. Ethnicity

Eleven studies found differences between ethnicities such as a higher risk in aboriginal ethnicity (IRR 1.93; 1.63–2.28) [13] and HR 1.78; 1.45–2.19 [39], Maoris (OR 1.64; 1.43–1.87) [13], First Nation Canadians (*P* = 0.012) [16], and black ethnicity in comparison to Caucasians (HR 1.255; 1.178–1.338, IRR 2.2; *P* < 0.01; HR 1.5; 1.2–1.8; IRR 1.629; *P* = 0.004; and IRR 1.37; 1.00–1.88) [14, 28, 30, 37, 40]. Lim et al. [15] reported not only an increased risk for peritonitis in indigenous people who lived far away from their treatment center (“remote”), but also a higher risk for technique failure, all-cause and peritonitis-related mortality. African Americans also had a higher risk for peritonitis (IRR 1.36; 1.04–1.77) [20]. A significantly shorter time to first episode of peritonitis has been observed in Native Canadians (*P* < 0.01) [34]. In contrast, Troidle et al. [32] showed that white people did not have a significantly decreased risk for peritonitis compared to other ethnicities (HR 0.90; 0.39–2.35). Furthermore, Nessim et al. [40] did not detect an increased risk for Asian people (IRR 0.89; 0.74–1.08).

Although these studies adjusted for some psychosocial factors and/or socioeconomic status in multivariable analyses, residual confounding might also account for these findings. Furthermore, it is possible that this association reflects a lower ability of receiving social support or health care service in these patient groups. Whether social assistance might decrease the risk for peritonitis in certain ethnicities remains unknown. 

#### 3.1.2. Age

Results on age as a risk factor were inconsistent. Four studies found an increased risk for peritonitis in older patients defined as >65 or >70 years [8, 12, 13, 17] whereas two studies which were both conducted in almost the same patient cohort could not confirm this association [7, 10] as well as three other studies did not detect an association between age and the risk for peritonitis [23, 32, 39]. Interestingly, one study conducted in a large patient cohort of 11975 subjects even found a higher risk in patients under the age of 65 [14] as well as the study by Zent et al. did [28]. 

#### 3.1.3. Gender

Kotsanas et al. [12] found a significant increased risk for peritonitis in females (OR 1.91; 95% CI 1.20–3.01), whereas the large study register study by Oo et al. [14], including 11975 patients from the United States Renal Data System Database, did not find an increased risk in females. Furthermore, neither in the studies by Lobo et al. [17] nor Lim et al. [15] an increased risk for female patients could be detected. In a study by Wang et al. [20], risk difference between females and males did not reach statistical significance (IRR 1.25; 0.63–1.01 for females). On the other hand, Oygar et al. [42] could observe that the frequency of female patients was significantly higher in the patient cohort group who had multiple episodes of peritonitis (*P* = 0.01). 

#### 3.1.4. Comorbidities

Only a few studies evaluated the impact of mainly cardiovascular comorbidities on the risk for peritonitis. McDonald et al. [13] found an increased risk for peritonitis in patients with chronic lung disease (HR 1.1; 1.03–1.18) and in patients with coronary artery disease (OR 1.06; 1.01–1.12), whereas Oo et al. [14] detected an increased risk in patients with congestive heart failure (HR 1.101; 1.034–1.172). In a study by Lim et al. [15], patients with cardiovascular disease had a slightly increased risk for peritonitis (HR 1.09; 1.04–1.17) whereas patients with cerebrovascular disease did not have a higher risk (HR 1.04; 0.95–1.14). In another study by Lim et al. [39], patients without hypertension had a decreased risk for peritonitis compared to patients with hypertension (HR 0.76; 0.61–0.94). Oygar et al. [42] showed that anti-hepatitis C Virus Antibody positivity was significantly associated with the risk for peritonitis (OR 1.6; *P* = 0.03). Neither Troidle et al. [32] nor Viglino et al. [38] could show an impact of coronary artery disease or cardiovascular disease on the risk for peritonitis. On the other hand it has been shown in several case series and retrospective analyses that PD represents a safe and efficient alternative in patients with congestive heart failure [43]. 

#### 3.1.5. Diabetes Mellitus

Six studies [9, 10, 13, 14] found an increased risk for peritonitis in diabetic patients compared to non-diabetics. Hazard ratio for type 1 diabetic patients was 1.24 (1.08–1.42) and 1.10 (1.03–1.17) for type 2 diabetic patients [13] compared to non-diabetic patients. In mixed cohorts of type 1 and type 2 diabetic patients, hazard ratios were 1.131 (1.069–1.195), 1.50 (1.05–2.40), and 1.64 (1.08–2.50), respectively [9, 10, 14]. In one study [28] a significantly increased risk for peritonitis was observed in patients with diabetes (IRR 1.81; *P* < 0.001). Interestingly, Nessim et al. [40] could only observe an increased risk for female diabetic (IRR 1.27; 1.10–1.47) but not for males (IRR 0.99; 0.87–1.13). In contrast, six studies [7, 15, 27, 32, 33, 35] could not show an association between diabetes and the risk for peritonitis. 

As diabetes mellitus is regarded as a risk factor for infections in general [44], it seems to be reasonable to consider it also as a risk factor for peritonitis in PD patients. Nevertheless, none of these studies provided mean or median HbA1c levels, fasting plasma glucose, or detailed information on treatment for diabetes. Hence, whether the diagnosis of diabetes itself or insufficient control of blood glucose levels are the basis for these findings remains unclear. As diabetic nephropathy is the leading cause of chronic renal failure in the United States and in Western countries diabetes as a risk factor for peritonitis requires attention but should definitely not be considered as a contraindication for PD treatment. It should be evaluated in further studies whether intensified glucose control in diabetic patients on PD could decrease the risk for peritonitis. 

#### 3.1.6. Underlying Renal Disease

Huang et al. [24] showed that patients with lupus nephritis as underlying renal disease had a significant increased risk for peritonitis (*P* < 0.02). Unfortunately, HR was not reported. Whether this association is contributed to the use of steroids or lupus nephritis itself remains unclear. There is only study which evaluated the impact of immunosuppression on the risk for peritonitis [35]. Thus, doubts about the true value of this factor remain. Glomerulonephritis as underlying disease was borderline significantly associated with a decreased risk for peritonitis in a study by Nessim et al. [40] (IRR 0.87; 0.75–1.00). 

#### 3.1.7. Residual Renal Function

Han et al. [9] found a HR of 0.81 (95% CI 0.74–0.88) per 1 mL/min/1.73 m² increase in residual GFR in reducing the risk of peritonitis. The authors state that their finding of residual renal function as a protective factor could at least partially be mediated by the better preserved nutritional status. Another possible explanation might be that patients with residual renal function have to perform fewer bag changes per day which might decrease the risk for peritonitis.

### 3.2. Modifiable Risk Factors (Table 4)

#### 3.2.1. Malnutrition

In three studies [17, 20, 31], albumin levels <3 g/dL or <2.9 g/dL, respectively, were associated with an approximately two-fold risk for peritonitis. Three studies showed an association between low albumin levels and a higher risk for peritonitis (HR 1.67; 1.08–2.60 per 10 g/L decrease [10], HR 0.73; 0.59–0.91 per 1 g/dL increase [11], and OR 1.2 (*P* = 0.05) per 1 mg/L [42], resp.). One study by Ozturk et al. [22] found a significant increased risk for subsequent peritonitis when albumin levels were declining. However, in three studies the association between low levels of albumin and the risk for peritonitis could not be confirmed [7, 9, 25]. One study [21] described a significant decreased risk for peritonitis in patients without malnutrition assessed by Subjective Global Assessment (HR 0.08; 0.018–0.365). It might be hypothesized that hypoalbuminemia, as a result of malnutrition, inflammatory response, or of uremia itself, may lead to a higher susceptibility to infection. Furthermore, the association between low levels of albumin and a subsequently higher risk for infections has also been established in patients on hemodialysis almost twenty years ago [45]. The finding that malnutrition and the risk for peritonitis may be associated is of special interest because a great proportion of patients is malnourished at the initiation of PD treatment [21]. However, diabetes where shown to increase the risk for peritonitis-related death in a study by Han et al. [9], whereas a higher residual renal function was identified to be a protective factor, thus implicating the importance of preventing or correcting malnutrition in PD patients. Further studies evaluating the impact of correcting malnutrition on peritonitis rate, morbidity, and mortality are clearly needed.

#### 3.2.2. Overweight

In a large evaluation by McDonald et al. [13] an increased risk for peritonitis was found with increasing body mass index (HR 1.08; 1.04–1.12 per 5 kg/m^2^), as well as Lim et al. [39] showed that a BMI > 30 kg/m^2^ was significantly associated with a higher risk for peritonitis (HR 1.25; 1.04–1.50), whereas in patients with a BMI < 20 or between 25–29.9 kg/m^2^ the risk for peritonitis was not significantly different from that in patients with a BMI between 20 and 24.9. In another study by Lim et al. [15], patients with a BMI > 30 kg/m^2^ were compared to patients with a BMI ≤ 18.5 and had an increased risk for peritonitis (HR 1.21; 1.01–1.43). In a study by Chow et al. these results could not be confirmed [10]. It might be hypothesized that there might be an association between high BMI and peritonitis through colonization and infection of PD catheters shortly after their insertion, resulting from increased wound area, reduced resistance of fat to infection, accentuated abdominal wall trauma stemming from a need for more vigorous retraction, and an inability to obliterate dead space in abdominal wall fat [10].

#### 3.2.3. Smoking

Kotsanas et al. [12] and McDonald et al. [13] showed an increased risk for peritonitis in current smokers, whereas in the study by Lim et al. [15] smoking was not associated with a higher risk (HR 1.04: 0.97–1.11). However, cigarette smoking affects both cell- and humoral-mediated immune responses [46, 47], thus implicating a plausible biological mechanism how the risk for peritonitis in current smokers might increase. Currently data on smoking as a risk factor remains inconclusive.

#### 3.2.4. *Staphylococcus aureus*

In a study by Luzar et al. [29] no influence of nasal *Staphylococcus aureus*-carrier status on the risk for peritonitis of any cause was observed (*P* > 0.50). However, the risk for *Staph. aureus*-related peritonitis was increased in carriers. As a consequence, it seems to be reasonable to use topical mupirocin application in order to prevent peritonitis episodes caused by *Staph. aureus*.

#### 3.2.5. Comedication

Andrews et al. [35] observed a higher risk for peritonitis in patients with immunosuppression (*P* < 0.001). Two studies from our group [18, 19] investigated the impact of comedication on the risk for peritonitis in nearly the same patient cohort. The use of oral active vitamin D was associated with a significantly decreased risk for peritonitis (HR 0.20; 0.06–0.64) which might be explained by pleiotropic functions of vitamin D which include its involvement in induction and promotion of cell differentiation, inhibition of cell growth and immunomodulation. No significant effect on the risk for peritonitis could be observed in patients using Sevelamer as a phosphate-binder (HR 0.55; 0.21–1.42). Data on the association of comedication and the risk for peritonitis are scarce and the sample sizes of the evaluated studies [18, 19] are low. 

#### 3.2.6. Psychosocial Factors

Two studies [26, 32] evaluated the influence of depression on the risk for peritonitis. The first study found an almost three-fold increased risk for peritonitis in patients with depression (HR 2.70; 1.23–6.03), the latter one found a significant difference in rates of peritonitis in patients with depression compared to those without (*P* < 0.05). These findings are of high interest since recent studies showed that approximately 20–30% of ESRD patients suffer from major depression (reviewed in [48]). Unfortunately, precisely in retrospective studies which comprise the majority of studies on risk factors for peritonitis it is almost never feasible to accurately identify those patients. Another study by Zent et al. [28] reported an increased risk for peritonitis in patients with passive dependent personality. 

#### 3.2.7. Socioeconomic Status

Farias et al. [37] observed a higher risk in patients with substance abuse (HR 1.9; 1.1–3.2) and in patients who lived in a rented house (HR 1.2; 1.0–1.5). Chow et al. [7] found an increased risk forilliterate patients (HR 2.73; 1.04–7.20) and people receiving social security assistance (HR 2.69; 1.10–6.54). In one study by Lobo et al. [17], an educational level of <4 years of schooling was associated with a two-fold increased risk for peritonitis (OR 2.15; 1.09–4.24) as well as in a study by Korbet et al. [30], a significantly decreased risk was detected per year of education (IRR 0.945; *P* = 0.028). However, the evidence on this topic is scarce. Chow et al. [7] state that they cannot exclude the possibility that their findings on social factors which increased the risk for peritonitis significantly were an indirect measure of depression in the examined patients.

#### 3.2.8. Patient's Choice

Three studies found an influence of patient's choice on the risk of peritonitis. In a study by Viglino et al. [38], patients who performed PD only as second choice treatment had a significantly shorter time to first peritonitis (RR 1.32; *P* < 0.001). Rodríguez-Carmona et al. [36] showed that patients who performed PD against their choice or their first physician's choice had a 1.6-fold increased risk for peritonitis (HR 1.6; 1.1–2.2) as well as Oygar et al. [42] showed that these patients had an increased risk (OR 2.6; *P* = 0.04). These patients mainly presented with contraindications such as poor personal or social conditions, complicated by an inability to obtain an adequate vascular access, and presumed/confirmed hemodynamic instability on hemodialysis.

#### 3.2.9. Former Modality

Nessim et al. [40] could show that transfer from HD (IRR 1.24; 1.11–1.38) was associated with a higher risk for peritonitis whereas starting PD after a failed transplant was not (IRR 1.27; 0.95–1.69).

They hypothesize that this increased risk may be attributable to two high-risk groups: those who were “crash starts” on HD with little predialysis care who subsequently chose to transfer to PD, and those who had been on HD for years and were out of vessel. 

## 4. Discussion

In 2007, Chow and Li [49] published a narrative review on risk factors for peritonitis, using the terms “modifiable” and “nonmodifiable” risk factors. Although it is sometimes hard to decide whether a risk factor could be modifiable, we adopted this approach, added a methodological quality scoring and updated the existing literature. One limitation might be that only one database had been used and therefore studies have been missed but on the grounds to provide a literature overview rather than a meta-analysis we think this is justified. Unfortunately, the comparability of the examined studies is limited due to highly varying patient selection in centers, countries, and even continents and differences in clinical practice. For example, diagnosis of peritonitis was established according to different guidelines in several centers, and exclusion criteria for episodes of peritonitis varied to a great extent, ranging from analyzing only the first episode of peritonitis and exclusion of relapses of established episodes to exclusion of all episodes of sterile peritonitis in patients using icodextrin. However, overall evidence was passable with nearly 80% of studies being scored as having acceptable methodological quality. From initially 415 identified abstracts, only 44 full-text articles were scored for their methodological quality. The other studies had to be excluded because they did not evaluate risk factors related to the individual (21 studies), reported on peritonitis episodes caused by a special group of germs (e.g., studies evaluating risk factors for fungal peritonitis; 128 studies), six studies evaluated cohorts including children, five studies evaluated very small patient cohorts <40 patients and one center reported on a single event leading to an outbreak of peritonitis.

In summary plenty of risk factors for peritoneal dialysis-associated peritonitis have been identified in studies of acceptable methodological quality. However, the evidence for many of these risk factors is based on single studies or studies including a relatively small patient number. Diabetes mellitus, ethnicity, and malnutrition might be considered as relatively well-established risk factors for peritonitis. Data on the impact of comorbidities are scarce. Whether the presence of multiple identified risk factors in an individual should lead to the definition of a “high risk patient” has not been evaluated yet. However, it seems reasonable to hypothesize that patients presenting with a number of these identified patient risk factors, might be at higher risk for peritonitis. Furthermore, it remains elusive if modification of one or more of these risk factors would result in a reduction of the peritonitis rate and probably in a higher rate of technique survival in PD patients. Nevertheless, the decision whether a patient with certain risk factors should perform PD remains the choice of the individual patient and the treating nephrologist. However, data from this and from other reviews might serve as a basis to score patients as low and high risk, and thus facilitate the short- and longterm management of these patients. 

## 5. Conclusion

Data on modifiable and nonmodifiable risk factors for peritonitis are limited. Nevertheless, available evidence might be used as a basis for patient selection for peritoneal dialysis, and also for the grade of monitoring of high-risk patients. Especially diabetes mellitus, ethnicity and malnutrition might be considered as relatively well established risk factors for peritonitis. Nevertheless, due to the somewhat limited quality of the available evidence the decision whether a patient with certain risk factors should perform PD remains the choice of the individual patient and the treating nephrologist.

## Figures and Tables

**Table 1:** Assessment of methodological quality. Each statement scored with one point for the quality scoring.

(1)	Provide in the abstract an informative and balanced summary of what was done and what was found.
(2)	State specific objectives, including any prespecified hypotheses.
(3)	Describe the setting, location, type of data collection and relevant dates, including periods of recruitment.
(4)	Describe relevant data of follow-up time, including end of study period.
(5)	Give the eligibility criteria of participants, and the sources and methods of selection.
(6)	Clearly define all outcomes, exposures, predictors, potential confounders, and effect modifiers. Give diagnostic criteria for episodes of peritonitis.
(7)	Explain how the study size was arrived at.
(8)	Describe all statistical methods, including those used to control for confounding.
(9)	Describe any methods used to examine subgroups and interactions.
(10)	Give demographic characteristics of study participants, at least gender and age.
(11)	Summarize follow-up time (average per patient and total amount).
(12)	Report numbers of peritonitis episodes or peritonitis rate over time.
(13)	Give unadjusted and confounder-adjusted estimates and their precision.
(14)	Discuss limitations of the study, taking into account sources of potential bias or imprecision.
(15)	Give a cautious overall interpretation of results considering objectives, multiplicity of analyses, results from similar studies, and other relevant evidence.

**Table 2:** Characteristics of identified studies on patient-based risk factors for PD-associated peritonitis.

Study	Number of patients	Age (years)	Female (%)	FU-time (months)	Ethnicity	Peritonitis rate	CAPD/APD
[7]	102	57.0 ± 13.0	38.2	10.7^a^	Asian	0.36/patient year	Both
[8]	149	62.2 ± 5.3	41	33 ± 27	N.R.	N.R.	Both
[9]	204	54.0 ± 11.5	42.6	37.5 ± 17.2	Asian	0.30/patient year	CAPD
[10]	246	51.0 ± 13	46	N.R.	Asian	0.48/patient year	APD
[11]	322	56.7 ± 12.5	45	23.9	Asian	4.63/100 patient years	CAPD
[12]	506	56.1 ± 15.3	49	N.R.	Mixed	N.R. for whole cohort	Both
[13]	10709	N.R.	49	N.R.	Mixed	0.86/patient year	Both
[14]	11975	58.8	46	24	Mixed	N.R.	Both
[15]	8237	59.9 ± 15.0	45.9	N.R.	Mixed	N.R.	Both
[16]	727	55.0 ± 14.8	44.7	N.R.	Mixed	N.R.	Both
[17]	330	53 ± 19	49.1	N.R.	N.R.	N.R.	Both
[18]	55	49.1 ± 13.5	43.6	23.6 ± 18.0	Caucasian	N.R.	Both
[19]	48	51.3 ± 14.3	41.7	25.0 ± 18.2	Caucasian	N.R.	Both
[20]	393	55.5 ± 15.0	39.7	13.4^a^	Mixed	N.R.	Both
[21]	56	56.2	28.6	20.8	N.R.	N.R.	Both
[22]	51	42.6 ± 14.3	53	N.R.	N.R.	N. R.	Both
[23]	54	50.3 ± 1.5	63	N.R.	Asian	N.R.	Both
[24]	69	34.2 ± 7.5	87	N.R.	Asian	N.R. for whole cohort	Both
[25]	71	43.3 ± 16.0	56	N.R.	Mixed	N.R.	Both
[26]	103	53.9 ± 13.0	45	12	N.R.	N.R.	Both
[27]	120	48.5 ± 15.0	33	N.R.	Mixed	N.R.	Both
[28]	132	42.4 ± 13.1	56	N.R.	Mixed	2.7/patient year	Both
[29]	140	56.4	33	10.4	N.R.	N.R.	Both
[30]	146	48.5 ± 15.0	46	N.R.	Mixed	N.R.	Both
[31]	147	43.6	41	N.R.	N.R.	N.R.	Both
[32]	162	55.4 ± 11.3	46	N.R.	Mixed	N.R.	Both
[33]	179	57.4 ± 12.3	54	N.R.	Asian	N.R.	Both
[34]	184	N.R.	N.R.	N.R.	Mixed	N.R.	Both
[35]	185	N.R.	48	N.R.	Mixed	0.8/patient year	Both
[36]	328	59.4 ± 15.7	47	20.9 ± 16.8	N.R.	N.R.	Both
[37]	1595	52.6 ± 15.0	46	N.R.	Mixed	N.R.	Both
[38]	1990	58.4 ± 14.8	44	24.2 ± 22.3	N.R.	0.68/patient year	Both
[39]	3162	N.R.	46	N.R.	Mixed	N.R. for whole cohort	Both
[40]	4247	N.R.	45	N.R.	Mixed	N.R.	Both
[41]	4247	59.0 ± 16.0	45	N.R.	Mixed	N.R.	Both

^a^Median. N.R.: not reported.

**Table 3:** Identified non-modifiable risk factors.

Ref	Risk factor	Statistics	Result	Meth. quality
Ethnicity				

[13]	Aboriginal ethnicity (versus non-indigenous ethnicity)	IRR (adj.)	1.93 (1.63–2.28)	Good
[39]	Aboriginal ethnicity (versus white)	HR (adj.)	1.78 (1.45–2.19)	Average
[15]	Indigenous and remote living (versus other)	HR (adj.)	1.92 (1.69–2.18)	Good
[16]	First Nations people (versus other)	Comp. of PET (not adj.)	*P* = 0.012	Good
[28]	Black ethnicity (versus other)	IRR (adj.)	2.2 (*P* < 0.01)	Average
[14]	Black ethnicity (versus white)	HR (adj.)	1.255 (1.178–1.338)	Good
[37]	Black ethnicity (versus white)	HR (adj.)	1.5 (1.2–1.8)	Average
[30]	Black ethnicity (versus white)	IRR (adj.)	1.629 (*P* = 0.004)	Average
[20]	African American (versus white)	IRR (adj.)	1.36 (1.04–1.77)	Average
[13]	Maori/Pacific Islander (versus non-indigenous ethnicity)	IRR (adj.)	1.64 (1.43–1.87)	Good
[34]	Native Canadian (versus Caucasian)	Time to first PE (not adj.)	*P* < 0.01	Average
[40]	Black ethnicity (versus other)	IRR (adj.)	1.37 (1.00–1.88)	Average
[32]	White ethnicity (versus other)	HR (adj.)	0.90 (0.39–2.35)	Average
[40]	Asian (versus other)	IRR (adj.)	0.89 (0.74–1.08)	Average

Age				

[12]	Age per 10 years	OR (adj.)	1.26 (1.07–1.48)	Good
[41]	Age per 10 years	IRR (adj.)	1.06 (1.01–1.10)	Average
[40]	Age per 10 years	IRR (adj.)	1.04 (1.01–1.08)	Average
[36]	Age per year	HR (adj.)	1.02 (1.01–1.03)	Average
[14]	Age from 45–64 (versus 65–74)	HR (adj.)	1.094 (1.007–1.188)	Good
[13]	Age from 65–74 (versus 45–54)	HR (adj.)	1.14 (1.06–1.22)	Good
[13]	Age from 75-84 (versus 45–54)	HR (adj.)	1.28 (1.15–1.43)	Good
[17]	Age ≥ 65 years (versus <65 years)	OR (adj.)	2.15 (1.09–4.24)	Good
[8]	Age > 70 years (versus <70)	Comp. of PET (not adj.)	*P* < 0.002	Good
[13]	Age > 85 years (versus 45–54)	HR (adj.)	1.94 (1.20–3.13)	Good
[14]	Age < 45 years (versus 65–74)	HR (adj.)	1.094 (1.007–1.188)	Good
[28]	Younger age (NFI)	IRR (adj.)	N.R. (*P* < 0.008)	Average
[32]	Age ≥ 65 years (versus <65)	HR (adj.)	0.80 (0.29–1.48)	Average
[14]	Age ≥ 75 years (versus <75)	HR (adj.)	1.071 (0.988–1.162)	Good
[39]	Age 0–24.9 years (versus ≥65)	HR (adj.)	0.90 (0.66–1.22)	Average
[39]	Age 25–44.9 years (versus ≥65)	HR (adj.)	0.83 (0.70–1.00)	Average
[39]	Age 45–64.9 years (versus ≥65)	HR (adj.)	0.88 (0.77–1.01)	Average
[10]	Age per year	HR (adj.)	0.99 (0.91–1.01)	Good
[7]	Age < 40 years	HR (adj.)	2.87 (0.80–10.30)	Good
[23]	Age ≥ 60 years (versus <60 years)	Time to first PE (not adj.)	*P* = 0.1704	Average
[38]	Age ≥ 65 years (versus <65 years)	Time to first PE (not adj.)	*P* = non-significant	Average

Gender				

[12]	Females (versus males)	OR (adj.)	1.91 (1.20–3.01)	Good
[14]	Females (versus males)	HR (adj.)	0.968 (0.918–1.020)	Good
[17]	Males (versus females)	OR (adj.)	0.73 (0.44–1.21)	Good
[15]	Males (versus females)	HR (adj.)	0.95 (0.89–1.02)	Good
[38]	Females (versus males)	Time to first PE (not adj.)	*P* = non-significant	Average
[20]	Females (versus males)	IRR (adj.)	1.25 (0.63–1.01)	Average

Comorbidities				

[13]	Chronic lung disease (versus no chronic lung disease)	HR (adj.)	1.10 (1.03–1.18)	Good
[14]	Congestive heart failure (versus no congestive heart failure)	HR (adj.)	1.101 (1.034–1.172)	Good
[13]	Coronary artery disease (versus no coronary artery disease)	IRR (adj.)	1.06 (1.01–1.12)	Good
[15]	Cardiovascular disease (versus no CVD)	HR (adj.)	1.09 (1.04–1.17)	Good
[39]	No hypertension (versus hypertension)	HR (adj.)	0.76 (0.61–0.94)	Average
[17]	Catheter exit site infection (versus none)	OR (adj.)	2.63 (1.57–4.41)	Good
[32]	Coronary artery disease (versus no coronary artery disease)	HR (adj.)	0.60 (0.39–1.79)	Average
[10]	History of cerebrovascular disease (versus no history)	HR (adj.)	1.39 (0.82–2.35)	Good
[15]	Cerebrovascular disease (versus no cerebrovascular disease)	HR (adj.)	1.04 (0.95–1.14)	Good
[38]	Cardiovascular disease (versus no cardiovascular disease)	Time to first PE (not adj.)	*P* = non-significant	Average

Diabetes mellitus				

[14]	Diabetes versus no diabetes	HR (adj.)	1.131 (1.069–1.195)	Good
[10]	Diabetes versus no diabetes	HR (adj.)	1.5 (1.05–2.40)	Good
[9]	Diabetes versus no diabetes	HR (adj.)	1.64 (1.08–2.50)	Good
[28]	Diabetes versus no diabetes	IRR (adj.)	1.81 (*P* < 0.001)	Average
[40]	Diabetes in females (versus no diabetes)	IRR (adj.)	1.27 (1.10–1.47)	Average
[13]	Type 1 diabetes (versus no diabetes)	HR (adj.)	1.24 (1.08–1.42)	Good
[13]	Type 2 diabetes (versus no diabetes)	HR (adj.)	1.1 (1.03–1.17)	Good
[35]	Diabetes versus no diabetes	Comp. of PET (not adj.)	*P* = non-significant	Average
[32]	Diabetes versus no diabetes	HR (adj.)	1.00 (0.46–2.17)	Average
[15]	Diabetes versus no diabetes	HR (adj.)	1.06 (0.94–1.18)	Good
[7]	Diabetes versus no diabetes	HR (adj.)	2.08 (0.88–4.95)	Good
[27]	Diabetes versus no diabetes	Time to first PE (not adj.)	*P* = 0.63	Average
[33]	Diabetes versus no diabetes	Time to first PE (not adj.)	*P* > 0.2	Average
[38]	Diabetes versus no diabetes	Time to first PE (not adj.)	*P* = non-significant	Average
[40]	Diabetes in males (versus no diabetes)	IRR (adj.)	0.99 (0.87–1.13)	Average

Underlying renal disease				

[24]	Lupus nephritis (versus other)	HR (adj.)	HR N.R. (*P* < 0.02)	Average
[40]	Glomerulonephritis (versus other)	IRR (adj.)	0.87 (0.75–1.00)	Average

Residual renal function				

[9]	GFR per mL/min/1.73 m^2^ increase	HR (adj.)	0.81 (0.74–0.88)	Good

Comp. of PET: comparison of peritonitis episodes per time period. HR: hazard ratio. NFI: no further information. IRR: incidence rate ratio. OR: odds ratio. PE: peritonitis episode.

**Table 4:** Identified modifiable risk factors.

Ref	Risk factor	Statistics	Result	Meth. quality
Malnutrition				

[11]	Albumin per g/dL increase	HR (adj.)	0.73 (0.59–0.91)	Good
[10]	Albumin per 10 g/L decrease	HR (adj.)	1.67 (1.08–2.60)	Good
[31]	Albumin < 3 g/dL (versus ≥3 g/dL)	Comp. of PET (not adj.)	*P* < 0.05	Average
[17]	Albumin < 3 g/dL (versus ≥3 g/dL)	OR (adj.)	2.03 (1.21–3.43)	Good
[22]	Declining Albumin	Comp. of PET (not adj.)	*P* = 0.026	Average
[21]	No malnutrition (versus malnutrition^a^)	HR (adj.)	0.08 (0.018–0.365)	Average
[20]	Albumin < 2.9 g/dL (versus ≥2.9)	IRR (adj.)	0.74 (0.61–0.89)	Average
[9]	Albumin per 1 g/dL increase	HR (adj.)	0.61 (0.37–1.13)	Good
[7]	Albumin per 10 g/L decrease	HR (adj.)	1.80 (0.68–4.80)	Good
[25]	Level of serum albumin	Comp. of RF (not adj.)	*P* = non-significant	Average

Weight				

[13]	BMI per 5 kg/m^2 ^	HR (adj.)	1.08 (1.04–1.12)	Good
[39]	BMI > 30 kg/m^2 ^(versus 20–24.9)	HR (adj.)	1.25 (1.04–1.50)	Average
[15]	BMI > 30 kg/m^2^ (versus 0–18.5)	HR (adj.)	1.21 (1.01–1.43)	Good
[39]	BMI < 20 kg/m^2^ (versus 20–24.9)	HR (adj.)	0.98 (0.81–1.20)	Average
[39]	BMI 25–29.9 kg/m^2 ^(versus 20–24.9)	HR (adj.)	1.08 (0.94–1.24)	Average
[10]	BMI per kg/m^2 ^	HR (adj.)	0.98 (0.91–1.05)	Good

Smoking				

[12]	Current smoking (versus never)	OR (adj.)	1.71 (1.04–2.82)	Good
[13]	Current smoking (versus never)	OR (adj.)	1.15 (1.07–1.23)	Good
[15]	Smoker (versus non-smoker)	HR (adj.)	1.04 (0.97–1.11)	Good

*Staph. aureus*				

[29]	*Staph. aureus*-carrier (versus non-carrier)	Comp. of PET (not adj.)	*P* > 0.50	Average

Comedication				

[18]	Use of oral active vitamin D (versus none)	HR (adj.)	0.20 (0.06–0.64)	Good
[35]	Immunosuppression (versus none)	Comp. of PET (not adj.)	*P* < 0.001	Average
[19]	Use of Sevelamer (versus none)	HR (adj.)	0.55 (0.21–1.42)	Good

Psychosocial factors				

[32]	Depression (versus no depression)	HR (adj.)	2.70 (1.23–6.03)	Average
[26]	Depression (versus no depression)	Comp. of PET (not adj.)	*P* < 0.05	Average
[28]	Passive dependent personality (NFI).	IRR (adj.)	N.R	Average
[37]	Substance abuse (versus no substance abuse)	HR (adj.)	1.9 (1.1–3.2)	Average

Socioeconomic status				

[30]	Education per year	IRR (adj.)	0.945 (*P* = 0.028)	Average
[17]	Educational level < 4 years of schooling (versus ≥4 years)	OR (adj.)	2.15 (1.09–4.24)	Good
[37]	Student (versus no student)	HR (adj.)	2.4 (1.4–4.3)	Average
[7]	Illiteracy (versus literacy)	HR (adj.)	2.73 (1.04–7.20)	Good
[7]	Receiving social security assistance (versus no assistance)	HR (adj.)	2.69 (1.10–6.54)	Good
[37]	Living in a rented house (versus own house)	HR (adj.)	1.2 (1.0–1.5)	Average

Patient's choice				

[38]	PD as second choice (versus first choice)	Time to first PE (not adj.)	*P* < 0.001	Average
[36]	PD against patient's or first physician's choice	HR (adj.)	1.6 (1.1–2.2)	Average

Former modality				

[40]	Transfer from HD	IRR (adj.)	1.24 (1.11–1.38)	Average
[40]	Failed transplant (versus no failed transplant)	IRR (adj.)	1.27 (0.95–1.69)	Average

^a^Assessed by Subjective Global Assessment; BMI: body mass index. Comp. of PET: comparison of peritonitis episodes per time period. Comp. of RF: Comparison of levels of studied risk factor (peritonitis versus no peritonitis). HR: hazard ratio. OR: odds ratio. PE: peritonitis episode. *Staph. aureus*: *Staphylococcus aureus*. NFI: no further information.
